# Characterization of different bubble formulations for blood-brain barrier opening using a focused ultrasound system with acoustic feedback control

**DOI:** 10.1038/s41598-018-26330-7

**Published:** 2018-05-22

**Authors:** Chenchen Bing, Yu Hong, Christopher Hernandez, Megan Rich, Bingbing Cheng, Imalka Munaweera, Debra Szczepanski, Yin Xi, Mark Bolding, Agata Exner, Rajiv Chopra

**Affiliations:** 1 Department of Radiology, UT Southwestern Medical Center, Dallas, TX 75390 USA; 2 Department of Radiology, Case Western Reserve University, Cleveland, OH 44106 USA; 3 Division of Advanced Medical Imaging Research, University of Alabama, Birmingham, AL 35294 USA; 4 Department of Clinical Science, UT Southwestern Medical Center, Dallas, TX 75390 USA; 5 Advanced Imaging Research Center, UT Southwestern Medical Center, Dallas, TX 75390 USA

## Abstract

Focused ultrasound combined with bubble-based agents serves as a non-invasive way to open the blood-brain barrier (BBB). Passive acoustic detection was well studied recently to monitor the acoustic emissions induced by the bubbles under ultrasound energy, but the ability to perform reliable BBB opening with a real-time feedback control algorithm has not been fully evaluated. This study focuses on characterizing the acoustic emissions of different types of bubbles: Optison, Definity, and a custom-made nanobubble. Their performance on reliable BBB opening under real-time feedback control based on acoustic detection was evaluated both *in-vitro* and *in-vivo*. The experiments were conducted using a 0.5 MHz focused ultrasound transducer with *in-vivo* focal pressure ranges from 0.1–0.7 MPa. Successful feedback control was achieved with all three agents when combining with infusion injection. Localized opening was confirmed with Evans blue dye leakage. Microscopic images were acquired to review the opening effects. Under similar total gas volume, nanobubble showed a more reliable opening effect compared to Optison and Definity (p < 0.05). The conclusions obtained from this study confirm the possibilities of performing stable opening using a feedback control algorithm combined with infusion injection. It also opens another potential research area of BBB opening using sub-micron bubbles.

## Introduction

The blood-brain barrier (BBB) serves as a physiological barrier that separates the brain tissue and the circulation system. The BBB is comprised of endothelial tight junctions and upregulated drug efflux pumps that limits the delivery of large molecules and therapeutic agents into the central nervous system. Low pressure burst mode focused ultrasound (FUS) combined with microbubbles-based contrast agents has been shown to transiently open the BBB^1–4^. Trans-cranial delivery of the FUS energy enables localized BBB opening and a wide range of therapeutic molecules including chemotherapeutics^5,6^, viral vectors for gene therapy^7,8^, therapeutic agents for immunotherapy^9,10^, and natural killer cells^11,12^ have been successfully delivered into the brain after ultrasound exposure. However, it has been shown that the opening effect of FUS is highly dependent on the ultrasound exposure parameters^13–15^, injection rate of the agents^16^, dosage^14,17,18^, bubble formulation^19–21^, and other *in-vivo* factors such as oxygen levels and blood flow^22–24^. Consistent and stable BBB opening is highly desired for future clinical applications.

The conventional method to guide and confirm BBB opening is using magnetic resonance imaging (MRI)-guided focused ultrasound targeting followed by the acquisition of T1-weighted contrast enhanced images after treatment to detect leakage of contrast agent into the brain parenchyma^1,3,25,26^. A limitation of this method is the inability to monitor the treatment in real-time during ultrasound exposure to ensure sufficient energy being delivered to open the BBB while avoiding over-exposure of tissue and potential damage to the brain. The use of passive acoustic emission detection to monitor cavitation activity of bubbles during ultrasound exposure has been explored recently^27–32^. Stable cavitation caused by bubble oscillation can be identified by the detection of sub-/ultra-harmonic emissions^33^ while inertial cavitation leading to bubbles collapse and shock wave formation under higher focal pressure can be identified as broad-band energy in the acoustic emission spectrum^34^.

Fundamental studies about cavitation detection were primarily conducted to investigate various cavitation types and their influence on BBB opening effect. It is believed the BBB opening induced by FUS is mainly related to stable cavitation^27,31^ and inertial cavitation could serve as an indicator of tissue damage^35^. The threshold for inertial cavitation was determined by several groups^31,36^, and the effectiveness of using various frequency components in the acoustic emission spectrum as the indicator of BBB opening was evaluated. For an ultrasound transducer with the central frequency of F_0_, cavitation detection can be performed based on the fundamental frequency and its harmonics (n × F_0_, n = 1, 2, 3…)^37^ and its sub-/ultra-harmonics (n × F_0_, n = 0.5, 1.5, 2.5…)^32,38^. Combination of different frequency components were investigated as well^28^. In this study, we primarily focus on acoustic detection based on ultra-harmonics.

Intravenous administration of a bubble-based contrast agent is required to achieve BBB opening at low acoustic pressures. Currently there are several commercially-available microbubbles: Optison (GE Healthcare, Milwaukee, WI, USA), Definity (Lantheus Medical Imaging, North Billerica, MA, USA) and SonoVue (Bracco, Milan, Italy). These agents are approved by FDA for clinical diagnostic use and have also been confirmed to be effective in achieving BBB opening with FUS^7,27,31,39^. BBB opening effect was dependent on the circulation time of different bubble types, and also dependent on the bubble size and acoustic pressure. Hence, real-time monitoring of the acoustic emission and more stabilized bubble administration is highly desired.

To facilitate dissemination of ultrasound-mediated BBB opening to non-specialist fields outside of ultrasound, such as neurosciences, it is essential to produce reliable systems that can compensate for the variability in physical parameters described above, and which can integrate into standard neuroscience research platforms. Our group introduced a stereotactic focused ultrasound system for BBB opening in rodent subjects previously^40,41^ and characterized its ability to open the BBB. In this study we extend that prior work by integrating an acoustic hydrophone into the transducer in order to measure stimulated bubble emissions during pulsed ultrasound exposures. Infusion administration of microbubbles was also adopted instead of bolus injection to generate a more consistent bubble concentration in the bloodstream. The acoustic emissions from multiple bubble formulations (Optison, Definity and custom-made nanobubble) were characterized both *in-vitro* and *in-vivo*, and the feasibility of BBB opening under feedback control with these three bubble formulations was evaluated.

## Methods and Materials

### Focused ultrasound system and acoustic feedback control algorithm

**Figure 1:**
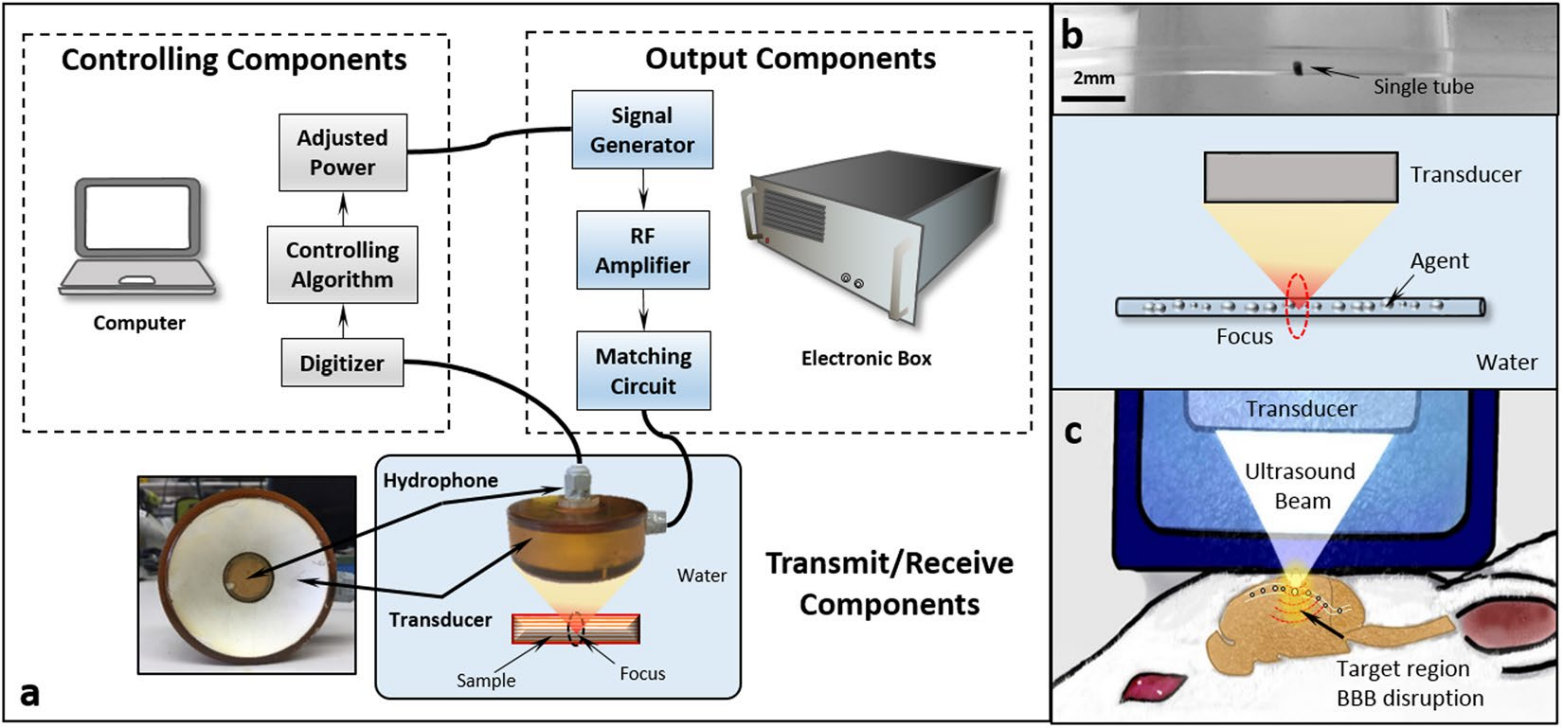
(**a**) Components of the acoustic feedback control system used in this study. (**b**) *in-vitro* tube phantom (**c**) *in-vivo* setup. The output signal to the transducer is controlled via computer. The acoustic emissions from stimulated microbubbles are measured with a confocal hydrophone and digitized by the computer. The frequency spectrum of the acoustic emissions is analyzed and the output signal to the transducer is adjusted based on the control algorithm. For the *in-vitro* study, a single tube phantom was used to evaluate the bubble response. A rat model was used for the *in-vivo* study.

The ultrasound system was adopted from our previous report^40^ and modified accordingly (see details in Supplementary Information). To evaluate the frequency response of acoustic emissions during BBB opening, a hydrophone was integrated into the center of the focused ultrasound transducer to capture the acoustic signals emitted by bubbles stimulated within the brain. The hydrophone was constructed from a flat 20-mm diameter PZT composite material (DL-54, center frequency 0.75 MHz, DeL Piezo Specialties, LLC, West Palm Beach, FL, USA), which was mounted in a 3D-printed housing and connected electrically to a 50 ohm coaxial cable. The signal acquired by the hydrophone was sampled at 20 MHz using a 14 bit PCI digitizer (ATS460, Alazar Technologies Inc, Pointe-Claire, QC, Canada) (Fig. 1a). Although the hydrophone was not focused, it was placed coaxial with the axis of the acoustic beam from the transducer, giving it a preferential sensitivity to signals coming from the focal region. No amplification of the hydrophone signal was required due to the strength of the measured emissions from bubbles.

**Figure 2:**
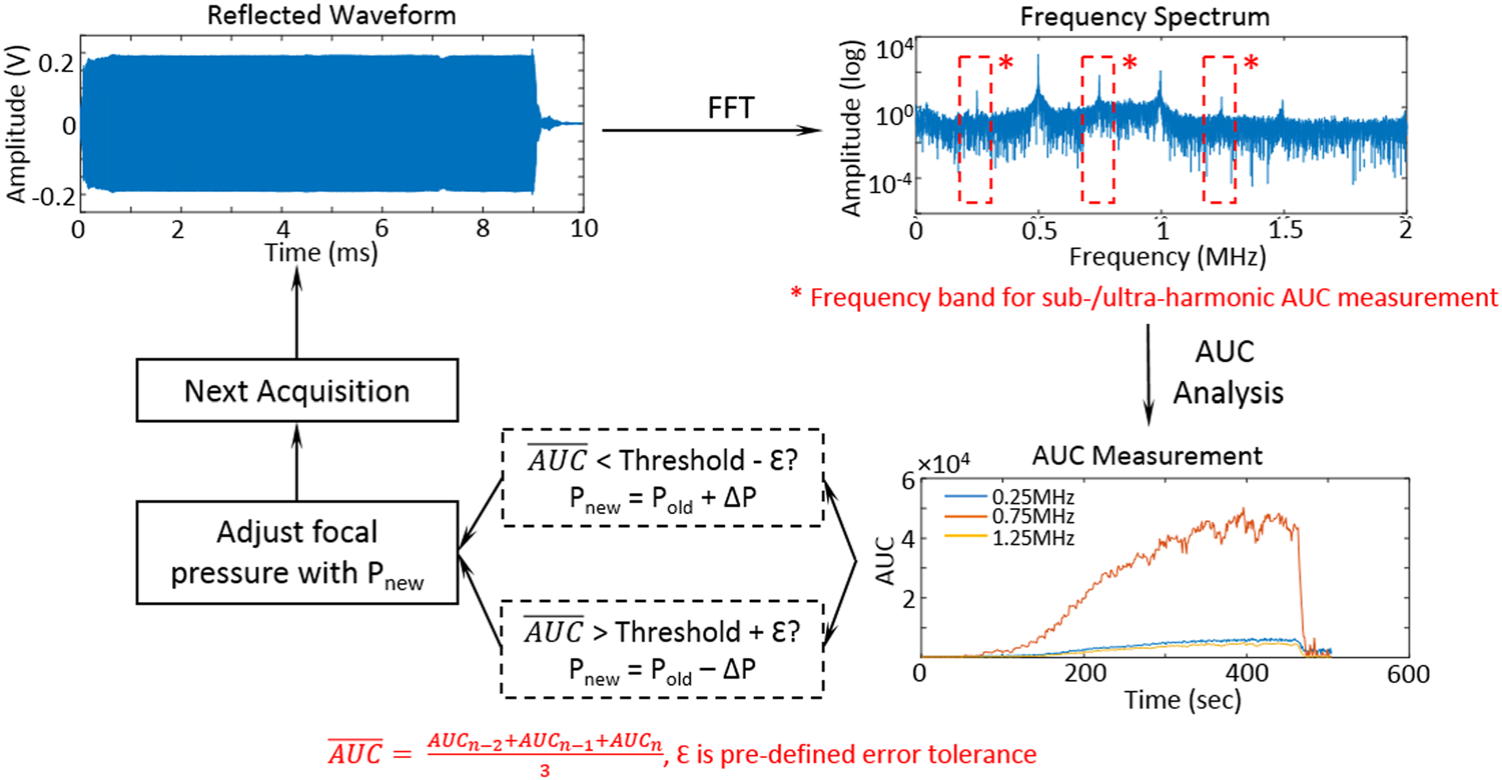
Flow chart for the feedback control algorithm. The digitizer captures the acoustic emissions from stimulated microbubbles during sonication, and a Fast Fourier transform (FFT) is then performed on the raw data. The area under curve (AUC) is calculated for sub-/ultra-harmonics (band width ± 0.05MHz). The mean AUC across 3 separate acquisitions is then compared to a target AUC threshold. If the measurement falls within a pre-defined error dead-band (Threshold ± ε), the pressure will be maintained, otherwise, a ±0.01–0.03 MPa adjustment will be applied to the transducer prior to the next acquisition. This algorithm runs continuously during the BBB exposure until the desired treatment time is reached.

The feedback control algorithm was implemented in LabVIEW (LabVIEW 2014, National Instruments, Austin, TX, USA). A flow chart of the algorithm is shown in Fig. 2. Each second a 10 ms burst was transmitted into the brain from the transducer in order to stimulate circulating bubbles. During each burst, a Fast-Fourier transform (FFT) was performed on the collected data, and the area under the curve (AUC) was calculated by summing the amplitude at sub- or ultra- harmonics within a ±0.05 MHz bandwidth (1,000 samples). Since the resonant frequency of the hydrophone was 0.75 MHz, this study mostly focused on the acoustic emissions at this frequency, which is the ultra-harmonic of the 0.5 MHz transmitting frequency (1.5 × F_0_). In order to improve stability, the AUC value that was input to the feedback algorithm was the average of the last three acquisitions. The average AUC was compared with a desired AUC threshold (AUC_targ_), and the pressure for the next transmission of ultrasound was determined. In addition, a dead-band, ε, defined as 0.2 × AUC_targ_, was applied to reduce the sensitivity to noise. If the AUC was within the range of AUC_targ_ ± ε the pressure level was not changed. For an AUC less or greater than AUC_targ_ ± ε, the pressure level was increased or reduced by 0.01–0.03 MPa. This process of measuring the AUC from acoustic emissions, comparing with the target threshold, and adjusting the pressure to maintain the AUC within the desired range, continued until the end of the prescribed exposure duration.

**Figure 3:**
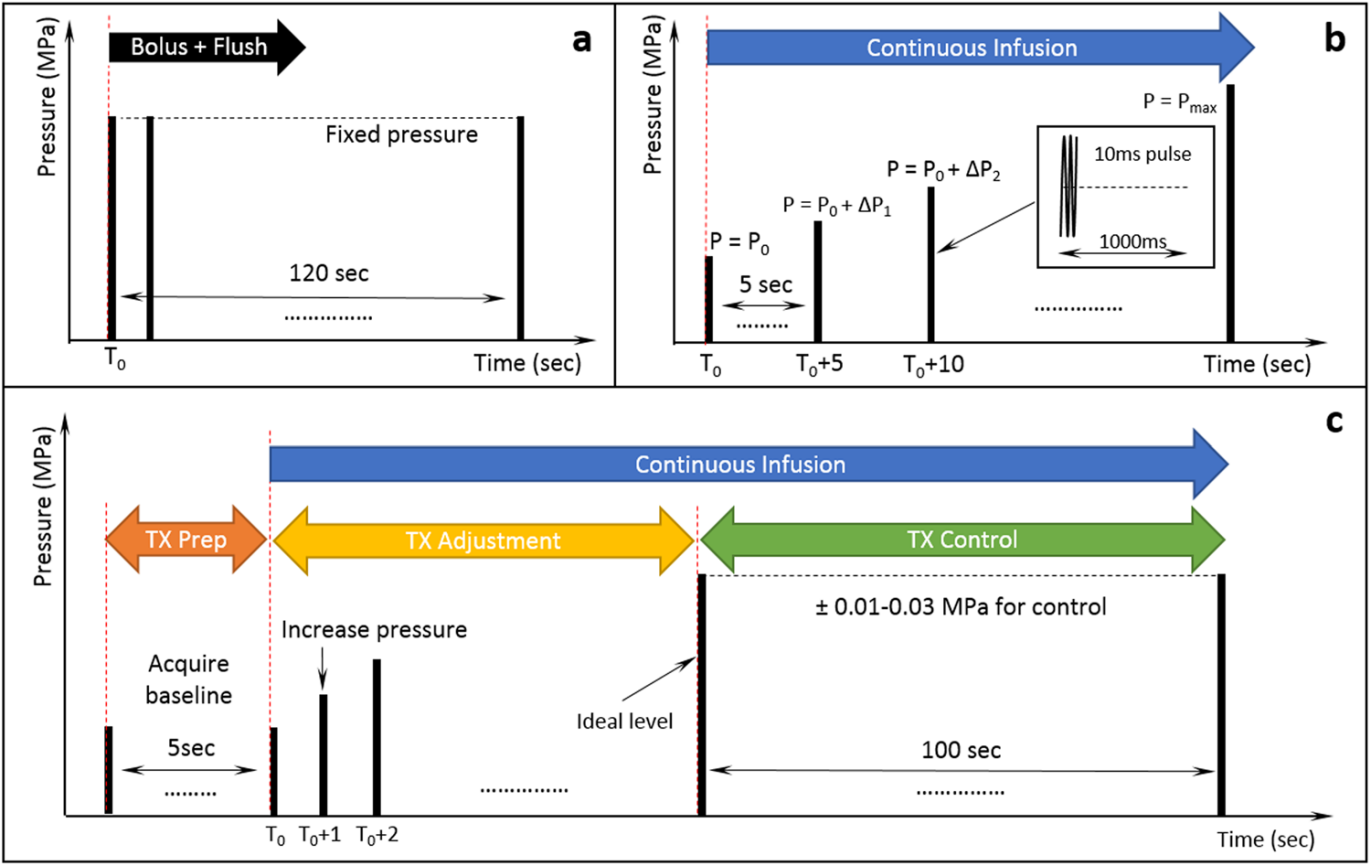
Three sonication protocols used in this study. (**a**) Fixed pressure sonication: A bolus of bubbles followed by a 0.2 ml saline flush were injected into the animal, with ultrasound exposures starting simultaneously. (**b**) Pressure Sweep: Bubbles were continuously infused from the beginning of the sonication with the transmit pressure level increased every 5 seconds in increments of 0.01–0.03 MPa. (**c**) Feedback controlled sonication: Bubble infusion started after a 5 second baseline acquisition. While bubble were continuously infused, the transmit pressure level was increased until the AUC response reached the target threshold. The pressure level was then adjusted to maintain the AUC at this level for 100 seconds to complete treatment. In all three sonication protocols the ultrasound transmit parameters were a 10 ms pulse and 1 Hz repetition rate.

Three sonication protocols were applied in this study: (A) constant pressure sonication, (B) pressure sweep, and (C) feedback controlled sonication. The flowchart of all three sonication protocols are shown graphically in Fig. 3. In the constant pressure sonication (Fig. 3a), ultrasound was delivered at a constant pressure and for a defined duration (120 seconds in a typical BBB opening experiment). This protocol was designed to evaluate the stability of various bubbles. The pressure sweep sonication (Fig. 3b) was used to observe the acoustic emission spectrum evolution with increasing pressure and determine the range of stable and inertial cavitation. Bubbles were infused through the sample volume during sonication, and the pressure was increased from 0.21 to 1.13 MPa in steps of 0.01–0.03 MPa. Up to 5 acquisitions were collected at each pressure level and the mean AUC and standard deviation across these measurements were recorded. For feedback controlled sonications (Fig. 3c), an initial 5 acquisitions were obtained prior to bubble infusion as a baseline reference. Once the infusion of bubbles started, the ultrasound pressure was increased until the measured AUC fell within the targeting range described above. At that point the pressure was adjusted for each pulse following the rules of the feedback algorithm until the total exposure time was reached. Feedback controlled sonication could be used to evaluate the feasibility of maintaining the stimulated bubble response at a fixed level and the corresponding opening effect.

### *In-vitro* characterization of three agents

#### Bubble description and preparation

Three bubble-based agents were evaluated in this study: Optison, Definity, and a custom-made nanobubble^42^. A brief description of the three agents is given in Supplementary Table S1. Optison (GE Healthcare, Milwaukee, WI, USA) consists 10 mg human albumin and 0.22 ± 0.11 mg/ml octafluoropropane in a 3 ml vial^43^. Definity (Lantheus Medical Imaging, Billerica, MA, USA) is perflutren lipid microsphere. After mechanical activation, a 2 ml Definity vial contains 0.75 mg lipid blend and 1.1 mg/ml octafluoropropane^44^. The nanobubble was prepared based on the method described in previous published articles^45^ (see the details in Supplementary Information).

In this study, the gas volume was kept constant across all samples of each bubble, in order to make comparisons of the frequency response of the different agents. According to the bubble size, volume of a single bubble (unit: µl) could be calculated using equation (1):

1 $${V}_{bubble}=\frac{4}{3}\pi {(\frac{d}{2})}^{3}\times {10}^{-9}$$

where *d* is the mean bubble diameter in µm. Minimum and maximum gas volume can be calculated based on the range of bubble size and bubble concentration. The mean gas volume for each agent is listed in Supplementary Table S1. To achieve a similar gas volume for all three agents, different dilutions were performed for each agent for all experiments in this study (Supplementary Table S2).

#### *In-vitro* frequency response characterization

A flexible tube with outer diameter of 1 mm (EXT-12HF, SAI Infusion Technologies, Lake Villa, IL, USA) was oriented across the ultrasound focus (Fig. 1b). For each experiment, the bubble solution was infused using an MRI compatible syringe pump (Chemyx NanoJet Stereotaxic Syringe Pump, Chemyx, Stafford, TX, USA) at a rate of 0.15 ml/min to refresh the bubble population within the focal volume of ultrasound after each pulse. All three sonication protocols were evaluated in the *in-vitro* study for each of the bubble formulations. Samples with five different gas volumes (listed in Supplementary Table S2) were characterized. The detailed characterization methods can be found in Supplementary Information.

#### *In-vitro* feedback control evaluation

The feasibility of maintaining the AUC of stimulated bubbles at a target level during sonication was first evaluated *in-vitro* prior to animal studies. Based on preliminary calibration experiments, an AUC controlling level of 5,000 was selected as it being the minimum consistent threshold with adequate signal-to-noise ratio (SNR). Bubble solutions of each agent were prepared with a gas volume of 1.1–1.2 µl/ml, and were infused into the flow tube via a syringe pump at a rate of 0.15 ml/min. The total sonication duration was 100 seconds and ultrasound power was adjusted after each pulse based on the difference between the measured AUC and the desired threshold.

### Animal studies

#### Animal preparation

Female rats (Sprague Dawley, 230–300 g, n = 40) were used in this study. All procedures were approved by UT Southwestern Institutional Animal Care and Use Committee and followed guidelines set forth by the Guide for the Care and Use of Laboratory Animals. Nine rats were used for primary *in-vivo* evaluation of the system. Twenty-five rats were used for *in-vivo* characterization and evaluation of three different agents and 6 rats were utilized for fluorescence microscopy. Detailed procedures are described in Supplementary Information.

#### Ultrasound exposure

The ultrasound setup for the animal study is shown in Fig. 1c. For bolus injection, Optison was injected with the dosage of 30 µl/kg. To facilitate accurate administration, the bubble solution was diluted with saline at a ratio of 1:20. To achieve a similar total gas volume (1.1–1.2 µl/ml) per dose for comparison, Definity was given at a dosage of 6 µl/kg with 1:100 dilution of saline. In the same manner, nanobubble was administrated as 737 µl/kg with 1:1 dilution. A constant pressure sonication was performed with the focal pressure to be 0.47 MPa calculated based on the insertion loss in rodents^46^. At least 5 minutes were allowed between two sonication for the bubbles to clear from circulation.

The infusion administration was controlled at 0.3 ml/min infusion rate using an MRI compatible syringe pump. The bubble solutions were prepared for three agents to reach a total gas volume of 1.1–1.2 µl/ml. For pressure sweep sonication, three acquisitions were acquired at each power level starting from 0.1 to 0.7 MPa. The duration of each power sweep sonication was approximately 125 seconds. For feedback controlled sonication, the desired threshold was set to be 5,000 for the ultra-harmonic of 0.75 MHz. This value was selected arbitrarily based on the previous characterization results as well as the consideration of signal-to-noise ration. The sonication duration for each target was approximately 100 seconds.

#### Microscopy

In order to assess brain tissue microstructure following BBB opening, after sacrificing the animal, brains were flash frozen and embedded in OCT compound (Sakuea Finetek, Torrence, CA) for cryosectioning. 10 µm serial sections were collected and stored at −80 °C until use. Micrographs of each whole brain slice were created by using stitching assistant software (NIS Elements, Nikon Instruments Inc, Melville, NY, USA) to combine adjacent 40x images. Details about microscopic imaging setups are included in Supplementary Information.

#### Statistical Methods

Statistic studies were performed on three aspects of the results to evaluate the difference among three bubbles in terms of ultrasound persistence, characteristics under pressure sweep, and the performances under *in-vivo* feedback control. All analysis was performed in SAS 9.4 (SAS Institute Inc., Cary, NC). Details for statistical studies are described in Supplementary Information.

## Results

### *In-vitro* characterization of three agents

**Figure 4:**
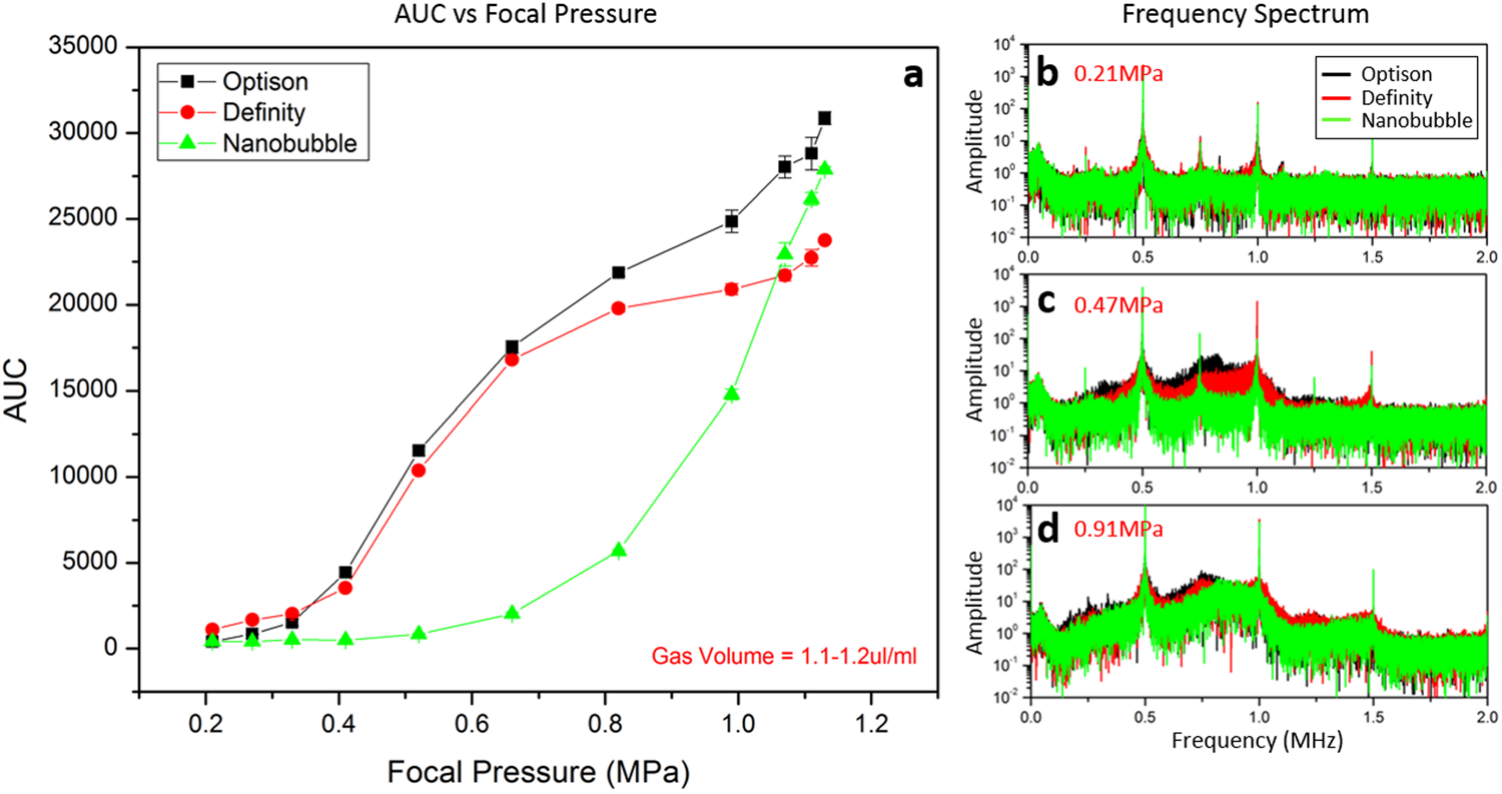
*In-vitro* characterization of three different agents: Optison, Definity, and nanobubble. (**a**) is the AUC response level as a function of focal pressure. For this characterization, all three agents were diluted to achieve a total gas volume of 1.1–1.2 µl/ml. (**b**–**d**) are frequency spectrums of three agents under various focal pressure. At a focal pressure of 0.21 MPa, discrete acoustic emissions at ultra-harmonics were detected indicating stable cavitation. At 0.47 MPa, broad-band emission was detected with Optison and Definity but not with nanobubble (**c**). Inertial cavitation was present at 0.91 MPa for all three agents (**d**).

Figure 4 shows the relationship between the frequency response (AUC) and focal pressure. Using samples with gas volume of 1.1–1.2 µl/ml, the detected AUC increased with higher focal pressure (Fig. 4a). At a maximum focal pressure of 1.13 MPa, the AUC level for Optison, Definity, and nanobubble was 31000 ± 370, 24000 ± 150 and 28000 ± 160 respectively. According to statistical assessment, the corresponding focal pressure level at the first break point is 0.37 MPa for Optison, 0.47 MPa for Definity and 0.59 MPa for nanobubble. Nanobubble required a higher power to trigger the rising phase compared to both Optison and Definity (p-value < 0.0001). The frequency spectrum at various focal pressures are included in panel b–d showing the transition from stable to inertial cavitation. At a lower focal pressure of 0.21 MPa (Fig. 4b), acoustic emission at the ultra-harmonic of 0.75 MHz was detected while with higher focal pressure of 0.47 MPa (Fig. 4c), broad-band emissions were observed for Optison and Definity, but not for nanobubble. As the focal pressure was increased to 0.91 MPa (Fig. 4d), all three agents showed broad-band emissions, which is believed to be an indicator of inertial cavitation.

**Figure 5:**
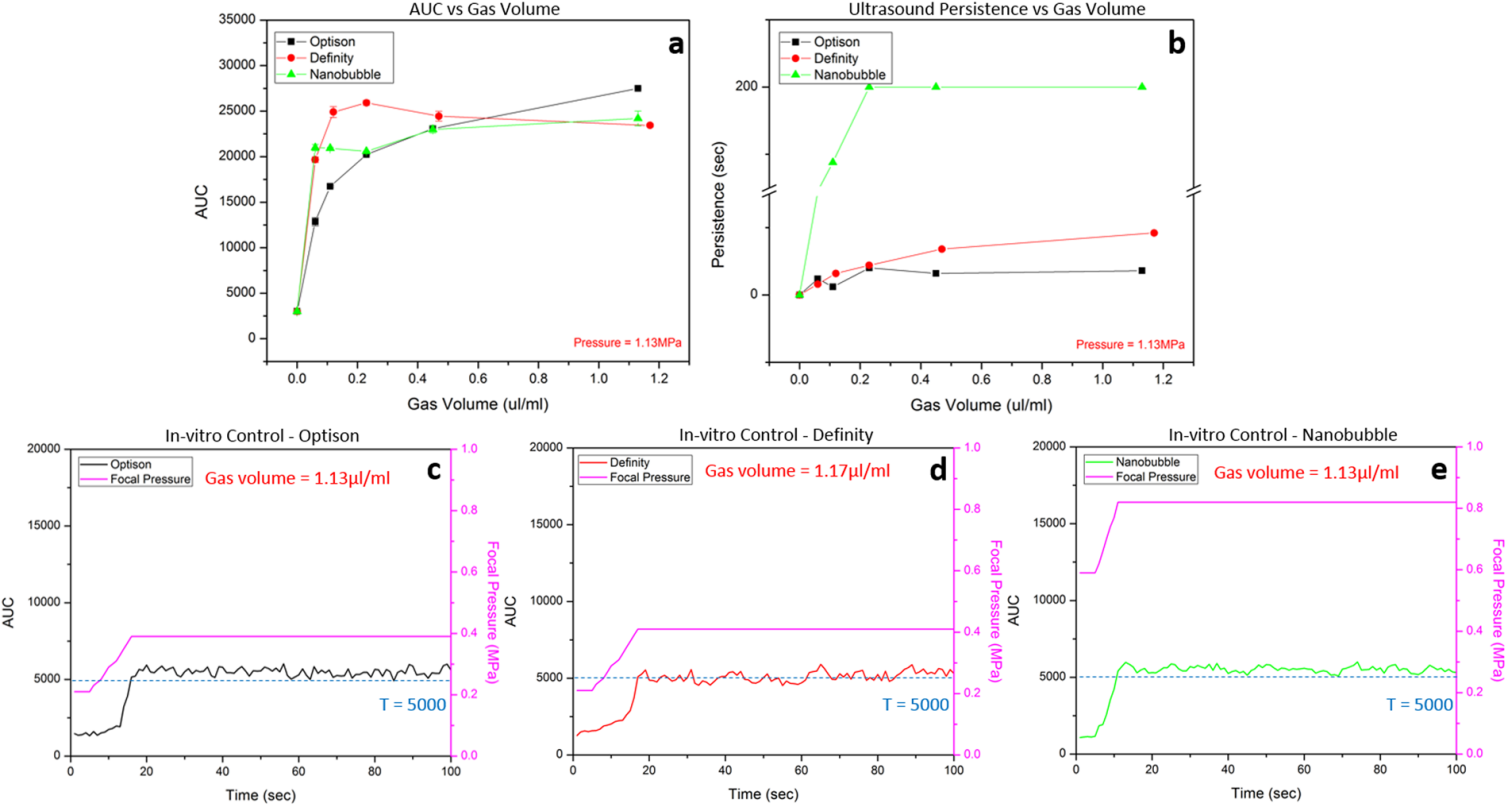
Characterization of the acoustic properties of three agents and their performance under feedback control. (**a**) The AUC response as a function of total gas volume for each agent. The AUC measured when sonicating degassed water was included as baseline control (gas volume = 0). (**b**) The persistence time of the AUC for different agents as a function of total gas volume. Nanobubble has better persistence compared to both Definity and Optison. All characterization results are based on the ultra-harmonic of 0.75 MHz. (**c**–**e**) Feedback control sonication in a single tube phantom using three agents: Optison (**c**), Definity (**d**) and nanobubble (**e**). The feedback control was performed on the ultra-harmonic of 0.75 MHz and the AUC control threshold was set as T = 5,000. All three agents were prepared to form a sample with total gas volume of 1.1–1.2 µl/ml. The pink curve indicates the focal pressure required to achieve the target AUC value.

Figure 5a,b shows the dependence of the AUC (0.75 MHz) and persistence on gas volume for all three agents. A higher AUC was observed for samples with a greater gas volume, although this reached a plateau above 0.5 µl/ml for Definity and nanobubble (Fig. 5a). Under the maximum focal pressure of 1.13 MPa, samples for Optison, Definity and nanobubble with less gas volume (0.06 µl/ml) presented the AUC level of 13000 ± 450, 20000 ± 310, and 21000 ± 340. On the other hand, with more gas volume (1.1–1.2 µl/ml) the AUC level was 27000 ± 100, 23000 ± 190 and 24000 ± 810, respectively. Interestingly, the AUC level of Definity started decreasing when the gas volume was larger than 0.23 µl/ml. Figure 5b evaluates the persistence of acoustic emissions as a function of gas volume. A longer persisted time was measured on samples with more encapsulated gas. For nanobubble samples with gas volume higher than 0.4 µl/ml, AUC level didn’t return to baseline value after 200 seconds, at which point data collection was terminated. Nanobubble had significantly longer persistence than both Optison and Definity at any gas volume (p-value < 0.0001), no significant difference was found between Optison and Definity (p-value > 0.2).

### *In-vitro* feedback control

The result of feedback control using the tube phantom is shown in Fig. 5c–e. Panel c–e are corresponding AUC and pressure curves for three agents respectively. With AUC_targ_ set at 5,000, the average AUC level across the control period for Optison, Definity, and nanobubble is 5490 ± 260, 5120 ± 330, and 5510 ± 240. The focal pressure required to maintain the AUC level at the AUC_targ_ was 0.41 ± 0.00, 0.39 ± 0.00 and 0.82 ± 0.01 MPa, respectively. Effective feedback control was confirmed *in-vitro*. For the *in-vitro* setup, the AUC could be maintained at the target value with very little change in pressure once the appropriate level was reached.

### *In-vivo* characterization of three agents

**Figure 6:**
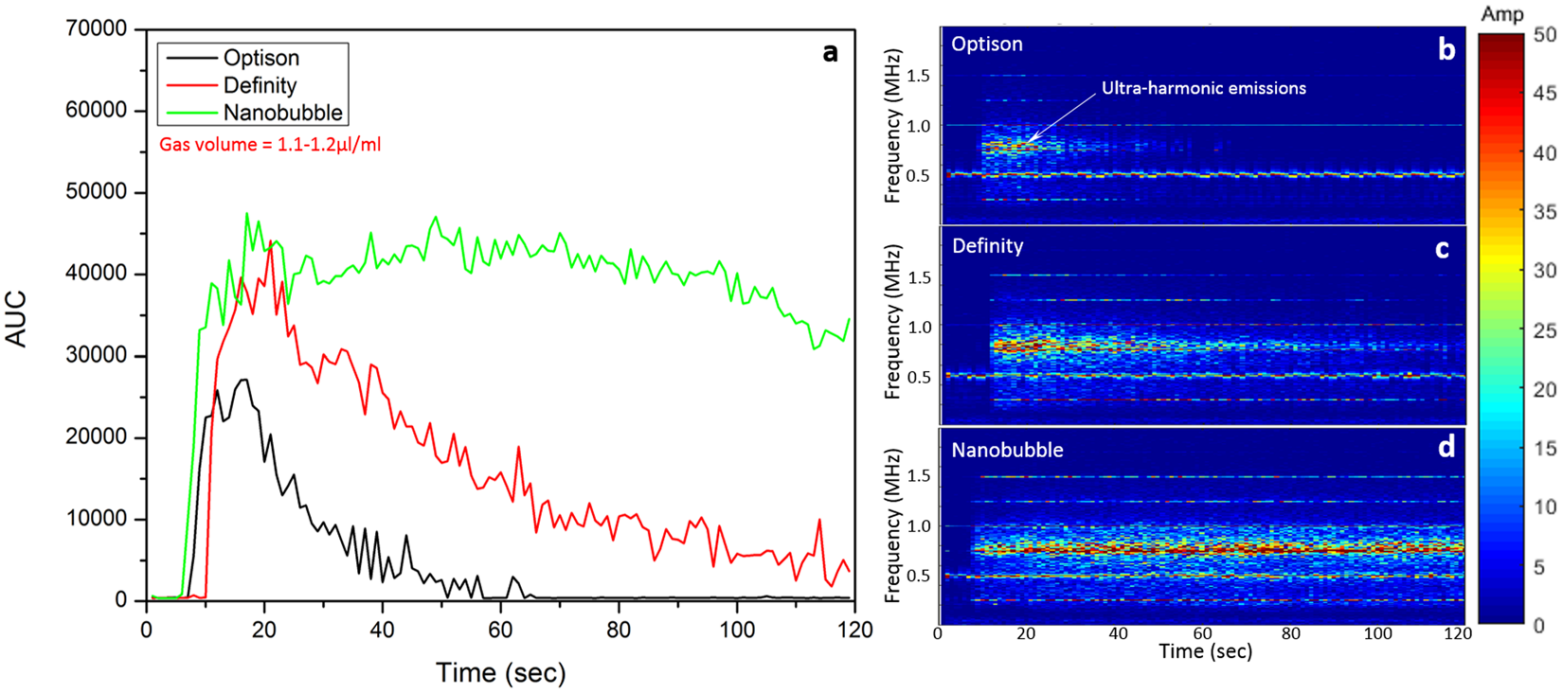
*In-vivo* ultra-harmonic acoustic response for 3 different agents after a bolus injection lasting 2 seconds. Ultrasound sonication started simultaneously with the injection of agent and lasted for 120 seconds. The three agents were diluted to form a sample with gas volume of 1.1–1.2 µl/ml. The final dose was then calculated accordingly to match the conventional dose for BBB opening in rodents. (**a**) The AUC at 0.75 MHz exhibited a significant increase followed by return to baseline for each of the three agents. (**b**–**d**) are the corresponding frequency spectrum for three agents respectively. The nanobubble exhibited the longest circulation time of all the agents, with an AUC still detectable after 120 seconds.

Figure 6 shows the AUC level observed during constant pressure sonication with bolus injection of three agents. Figure 6a shows the AUC over time and b–d are corresponding frequency spectrums over time shown as a spectrogram. Significant AUC increases were observed for all three agents shortly after the bolus injection, once bubbles reached the brain circulation. Definity and nanobubble had higher maximum AUC levels (39635.4 at 16 seconds for Definity, 47072.4 at 49 seconds for nanobubble) than Optison (27151.5 at 17 seconds). At the end of the sonication, the AUC level for Optison returned to baseline (AUC = 415.9) while weak frequency response still observed with Definity (AUC = 3654.9). A strong frequency response was still detected with nanobubble at the end of the sonication (AUC = 34576.4) indicating a longer *in-vivo* circulation time. The amplitude of acoustic emissions at ultra-harmonic of 0.75 MHz are shown in b–d. Continuous acoustic emissions across the entire sonication period were observed with nanobubble (Fig. 6d).

**Figure 7:**
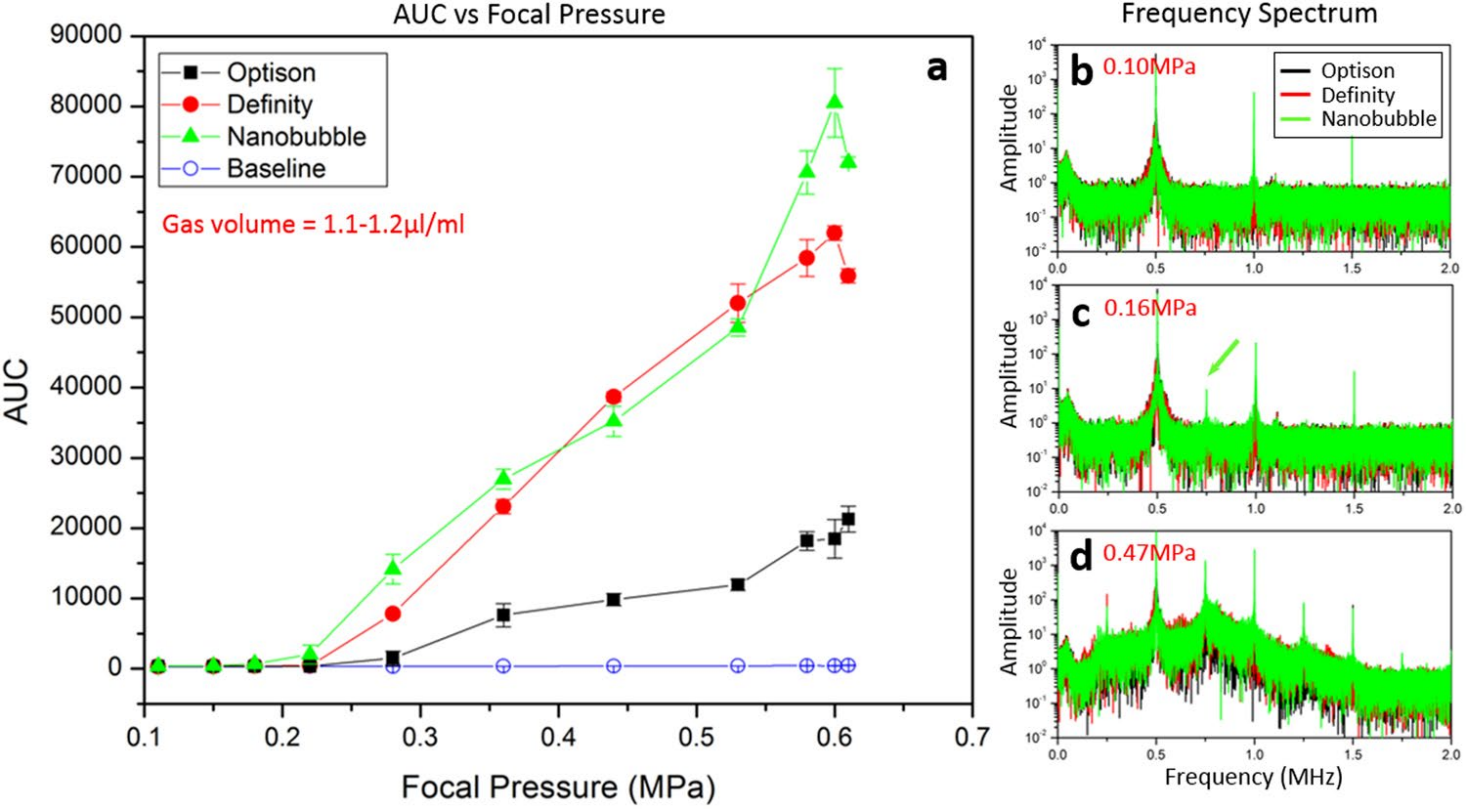
(**a**) The *in-vivo* AUC response for 3 different agents as a function of transmit focal pressure. All agents were prepared to form a sample with gas volume of 1.1–1.2 µl/ml. (**b**–**d**) The frequency spectrums for all three agents at different transmit pressures. At 0.15 MPa (**b**) no acoustic emission was detected at ultra-harmonic for all three agents. At 0.21 MPa (**c**), stable cavitation was detected (green arrow) for the nanobubble whereas no ultra-harmonic acoustic emission was detected with the other two agents. At a focal pressure of 0.47 MPa (**d**), inertial cavitation was detected for all three agents, represented as a broad increase to the background acoustic emission levels.

Figure 7 shows the *in-vivo* frequency response of three agents for a pressure sweep sonication. The AUC was plotted as a function of focal pressure, with a correction applied for insertion loss through the skull^46^ (Fig. 7a). All three agents showed AUC increases compared to baseline acquired prior to bubble infusion (maximum AUC = 453.5 ± 28.7). Definity and nanobubble showed higher maximum AUC (62000 ± 1000 at 0.6 MPa for Definity, 80000 ± 4900 at 0.6 MPa for nanobubble) than Optison (21000 ± 1800 at 0.6 MPa). Panel b–d are frequency spectrums acquired at different focal pressure indicating the presence of either stable cavitation or inertial cavitation. Under 0.10 MPa (Fig. 7b) no acoustic emission at ultra-harmonic was detected for all three agents (frequency amplitude < 0.5). At approximately 0.16 MPa (Fig. 7c), acoustic emission at 0.75 MHz was detected with nanobubble (frequency amplitude = 9.28) while not with other two agents. At 0.47 MPa (Fig. 7d), broad band emissions were detected with all three agents.

### *In-vivo* feedback control

**Figure 8:**
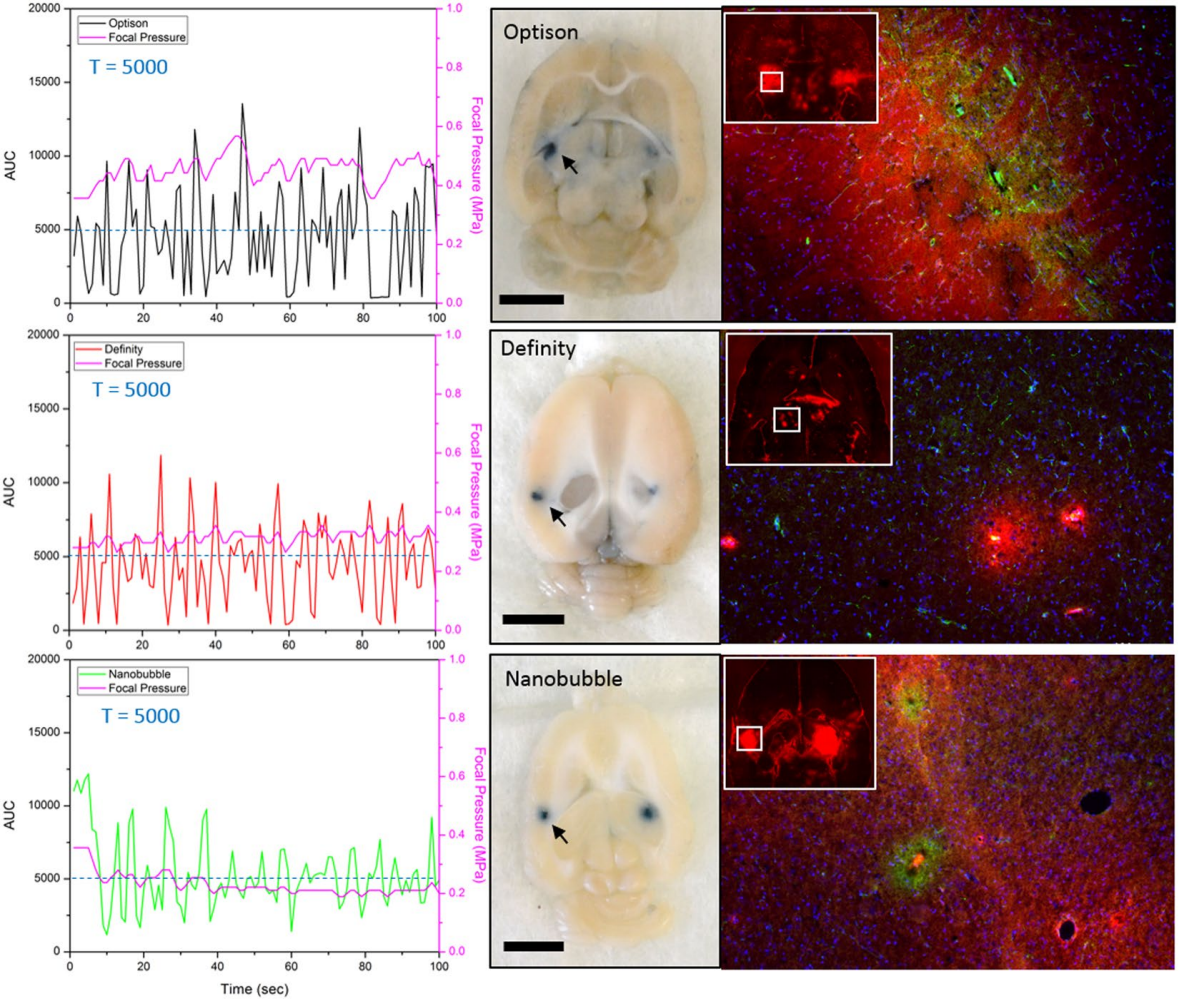
Demonstration of successful BBB opening with *in-vivo* feedback control based on the AUC responses for the 3 different agents. The left column shows the AUC as a function of time during a feedback control exposure with a target threshold of 5,000. The middle column is the white light dissection photo after the brain was harvested depicting localized Evans blue leakage in each hemisphere where the BBB was open (bar = 5 mm). The right column shows Evans blue distribution under fluorescence microscopy (red), RECA-1 immunofluorescence staining for endothelial cells (green) and DAPI nuclear stain for overall cellular morphology (blue).

A feedback-controlled sonication protocol was applied *in-vivo* with each of the three agents, with representative results shown in Fig. 8. The first column shows the AUC and delivered pressure during controlled treatment with each of the agents. With the controlling threshold set to be 5,000, the overall AUC level across all animals was 4690 ± 2745 for Optison, 4650 ± 3355 for Definity and 4870 ± 1853 for nanobubble. Transcranial focal pressure required to maintain the AUC level was 0.42 ± 0.04 MPa for Optison, 0.34 ± 0.03 MPa for Definity and 0.23 ± 0.02 MPa for nanobubble. Nanobubble had a significantly lower standard deviation of AUC than Optison (32% lower, p-value = 0.0019) and Definity (44% lower, p-value = 0.0002). It also required a lower focal pressure for control (0.19 MPa lower than Optison, p-value = 0.017 and 0.11 MPa lower than Definity, p-value = 0.0065). The variability of the required pressure (calculated as standard deviation of the required focal pressure) was also smaller for nanobubble compared to Optison (47% lower, p-value < 0.0001) and Definity (36% lower, p-value = 0.0147). Overall, nanobubble showed a better controlling performance compared to the other two agents.

### BBB opening effect with feedback controlled sonication

The second column in Fig. 8 shows block face photos of a brain dissection slice transverse to the ultrasound beam and at the level of the focus. Localized Evans blue dye leakage indicates BBB opening, and is present for all three agents. Immunofluorescent staining was performed to observe the degree of BBB opening and the cellular morphology of FUS targeted regions. The third column in Fig. 8 shows micrographs of 10-micron brain sections indicating the location of BBB opening with fluorescent Evans blue dye (red) in the target location. Regions of BBB opening were selected (indicated by white square) for imaging of endothelial cell morphology (green) as well as overall cellular morphology (blue). Overall cellular morphology appeared normal across regions of BBB opening however, endothelial cell changes were observed in regions of BBB opening compared to non-targeted regions. All three agents presented with endothelial cells that were wider in morphology and in the Optison and nanobubble groups the endothelial cells appeared more aggregated and uneven^47^ in regions of BBB opening compared to non-targeted regions. Furthermore, in the nanobubble and Optison groups, the morphological changes were more profound than in the Definity group which seemed to correspond to the degree of Evans blue dye leakage (red). Though further study is needed, these results suggest that bubble type may be sufficient to alter the effect of FUS on BBB cells and that RECA-1 maybe an attractive cellular marker for the degree of BBB opening in future studies evaluating the biological effects of BBB opening with feedback controlled sonication.

## Discussion

Ultrasound contrast agents are commonly used clinically in patients with suboptimal echocardiograms. When comparing the diagnostic performance of Definity, Optison and SonoVue^®^ in a preclinical model, no significant differences were detected among the different agents^48^. However, when being applied for BBB opening with focused ultrasound, differences in the acoustic characteristics as well as the stimulated emissions may impact how to best use each agent for this application. The acoustic behavior of bubbles depends on the properties of the surrounding medium, applied acoustic pressure, and the physical characteristics of the agent such as size distribution, internal gas, and shell type^49^. Wu *et al*.^50^ compared three contrast agents (Definity, SonoVue^®^ and USphere) with similar bubble dosage (4 × 10^7^ bubbles/kg), and found that the BBB opening effect can be predicable and bubble-independent given carefully controlled bubble dosage and administration. In our study, due to the significant variations in bubble size and concentration across the agents investigated, performing the characterization test with same number of bubbles was not considered a suitable control. Instead, we selected to compare their frequency response with similar total encapsulated gas in order to take both the bubble size and bubble concentration into consideration. These results might open another pathway for using different agents for BBB opening and drug delivery into the brain.

With a single tube phantom, the measured stimulated emissions with increasing pressure demonstrated a higher focal pressure was required to detect a harmonic response from the nanobubble (Fig. 4a). This result matches the finding from a previous study showing a higher acoustic pressure threshold is required with smaller bubble to achieve BBB opening^19^. As expected, samples with larger gas volume had a higher AUC response (Fig. 5a). Interestingly, each agent exhibited its own “saturation threshold” where the AUC no longer increased with increasing gas volume. For example, the maximum AUC level of Definity started to drop when the gas volume is larger than 0.23 µl/ml, while nanobubble started saturation with even lower threshold of 0.06 µl/ml. Under higher ultrasound exposures like shown in Fig. 5b (focal pressure = 1.13 MPa), bubble destruction impacts the persistence of the stimulated emissions^51,52^. Higher bubble concentration led to longer persistence from the bubbles, however the persistence time for the nanobubble was an outlier compared to the other agents (Fig. 5b). The exact mechanism for this increased persistence in still unclear, but may be related to the much increased number of bubbles for a given gas volume (due to the small size).

The *in-vivo* pressure sweep results were quite different from the *in-vitro* results. (Fig. 7a). Compared to Optison, Definity and nanobubble exhibited a much stronger *in-vivo* frequency response. Furthermore, the maximum *in-vivo* AUC levels for Definity and nanobubble were approximately 2–3 times higher than their *in-vitro* response. However, Optison maintained a similar AUC level both *in-vivo* and *in-vitro*. It is worth noting that the bubble size distributions are likely to evolve immediately after injection into the body^53^, which might account for some of these changes. Temperature change, *in-vivo* blood pressure and other factors such as gas flux might also contribute to alter the bubble distribution. The bio-distribution of these three agents might also be different, which is not addressed or controlled in this study. From the frequency spectrums acquired during the pressure sweep, a lower acoustic pressure level was required with nanobubble to observe ultra-harmonic activity (Fig. 7c), suggesting successful BBB opening can be achieved with nanobubble under relative lower focal pressure. This observation differed from the previous study performed in mice showing a higher acoustic pressure threshold was often required with smaller bubble^19^. One possible explanation is the nanobubble has a relatively higher concentration, certain amount of small nanobubbles may have a chance to combine with each other and form a cluster of bubbles with larger diameter compared to both Optison and Definity, but confirmation of this requires further investigation.

Previous studies have successfully implemented various feedback control algorithms to achieve BBB opening with focused ultrasound^28,32,50^. These studies, however, were mostly conducted with bolus injection where the systematic bubble concentration varies significantly after injection across a 2 minutes treatment. In our study, a steady infusion of bubbles was implemented for the first time. During bubble infusion, the focal pressure was adjusted in real-time to maintain the AUC level at a certain threshold, which means acoustic emissions were present throughout the entire treatment. This differs from protocols developed by others in which the focal pressure was reduced upon detection of sub- or ultra-harmonic emission to a level where these emissions were no longer present. Further study is required to determine whether the extent of BBB opening and impact on surrounding brain tissue is different from previous approaches.

Another limitation is the challenges and considerations for clinical translation of these concepts. First, the sonication duration required to achieve BBB opening at a particular AUC threshold should be carefully characterized. Second, the microbubble concentration required to obtain adequate signal to noise ratio from the acoustic receivers should be investigated. Last but not least, the system should have the ability to cover multiple targets in the brain to create a BBB opening over a larger region. The last point could be addressed with a phased array ultrasound system^54^. The other two factors, however, have not been fully evaluated yet.

The controlling threshold selected in this study (T = 5,000) was an arbitrary selection based on the characterization results and previously determined pressure thresholds for successful BBB opening^13^. This threshold also satisfied the adequate signal-to-noise ratio. However, a big limitation exists in this current setup is the lack of link between our selection of AUC value and the corresponding biological effect. Further studies to determine the appropriate controlling threshold to achieve BBB opening without causing damage to the surrounding tissue are required. The corresponding biological effect of various controlling threshold will be evaluated as well.

The robustness of feedback control algorithm was evaluated both *in-vitro* and *in-vivo*. Clear and stable control of acoustic emission levels at the ultra-harmonic was successfully achieved in the single tube phantom for all three agents (Fig. 5c–e). In the rat model, although successful BBB opening was achieved with all three agents using feedback control, the variability in acoustic emission levels during feedback controlled sonication varied a lot among three agents. One possible reason is the difference between experimental animals. The skull thickness, the size of the brain, and the vascular structure will result in an uneven spatial distribution of bubbles in the brain. Secondly, due to the possibility of different bio-distribution amongst the agents, using the same infusion rate for all three agents could result in different amount of bubble within the targeted region inside the brain. To perform reliable BBB opening with feedback control, the algorithm should be optimized for specific agents according to their acoustic properties and bio-distribution.

In BBB opening treatment with bolus injection, the circulation time plays a critical role especially during multiple spots sonications. Conventional multiple spot sonications were often performed in an alternating fashion where the transducer move between targets and return to its original location within one pulse^32,54–60^. In our study, the circulation time measured from the frequency response decay after a bolus injection (Fig. 6a) could serve as a valuable factor in optimize the sonication protocol. According to the *in-vivo* characterization results acquired in this study, nanobubble has a circulation time of approximately 6–8 minutes. This circulation time is much longer compared to both Optison and Definity (1–2 minutes). Previous studies from other groups have evaluated the effectiveness of multiple sonication after 1 dose of bolus injection^50^. An agent with longer circulation time will definitely simplify the treatment protocol. When combined with this specific nanobubble, FUS energy could be delivered across multiple spots without extra administration of agent. At the same time, this property will highly benefit the ability to cover a larger region without jeopardizing the treatment effect.

## Electronic supplementary material

Supplementary Information
