# Supplementary material for: Characterization of different bubble formulations for blood-brain barrier opening using a focused ultrasound system with acoustic feedback control
Source: Sci Rep. 2018 May 22;8:7986. doi: 10.1038/s41598-018-26330-7 (PMC5964106; doi:10.1038/s41598-018-26330-7)
Supplement: Supplementary file 1 — Supplementary Information [file 41598_2018_26330_MOESM1_ESM.docx]

**Characterization of different bubble formulations for blood-brain barrier opening using a focused ultrasound system with acoustic feedback control - Supplementary Information**

Chenchen Bing^1,*^, Yu Hong^1^, Christopher Hernandez^2^, Megan Rich^3^, Bingbing Cheng^1^, Imalka Munaweera^1^, Debra Szczepanski^1^, Yin Xi^1,4^, Mark Bolding^3^, Agata Exner^2^, Rajiv Chopra^1,5^

^1^ Department of Radiology, UT Southwestern Medical Center, Dallas, TX, USA

^2^ Department of Radiology, Case Western Reserve University, Cleveland, OH, USA

^3^ Division of Advanced Medical Imaging Research, University of Alabama, Birmingham, AL, USA

^4^ Department of Clinical Science, UT Southwestern Medical Center, Dallas, TX, USA

^5^ Advanced Imaging Research Center, UT Southwestern Medical Center, Dallas, TX, USA

*Corresponding author: Chenchen Bing, E-mail: chenchen.bing@utsouthwestern.edu, Department of Radiology, University of Texas Southwestern Medical Center, 5323 Harry Hines Blvd, Dallas, TX, USA 75390

**Table S1. Information for three tested bubble agents.**

| **Name** | **Formation** | **Size (µm)** | **# of bubbles/ml** | **Bubble volume (µl)** | **Gas volume (µl/ml)** | **Mean Gas volume (µl/ml)** |
| --- | --- | --- | --- | --- | --- | --- |
| Optison | perflutren protein | 3.0-4.5 | 5-8×10^8^ | 14.14 - 41.71×10^-9^ | 7.07 - 33.37 | 20.22 |
| Definity | perflutren lipid | 1.1-3.3 | 1.2×10^10^ max | 0.70 - 18.82×10^-9^ | 8.40 – 225.84 | 117.12 |
| nanobubble | perflutren lipid | 0.2-0.3 | 1×10^11^ | 0.004 - 0.014×10^-9^ | 0.40 – 1.40 | 0.9 |

**Table S2. Dilution information for three tested bubble agents.**

| **Name** | **Dilution** | **Gas volume (µl/ml)** |
| --- | --- | --- |
| Optison | 20, 50, 100, 200, 400 | 1.13, 0.45, 0.23, 0.11, 0.06 |
| Definity | 100, 250, 500, 1000, 2000 | 1.17, 0.47, 0.23, 0.12, 0.06 |
| nanobubble | 1, 2, 4, 8, 16 | 1.13, 0.45, 0.23, 0.11, 0.06 |

**Methods and Materials**

**Stereotaxic focused ultrasound system**

Ultrasound energy was transmitted into the brain using a focused transducer with a 75-mm diameter and a 60-mm radius of curvature. A custom-built hydrophone was inserted into a 26-mm diameter circular opening in the center of the transducer. The fundamental frequency of the transducer was 0.5 MHz as measured with an impedance analyzer (Via Bravo, AEA Technology Inc., Carlsbad, CA, USA), and an impedance matching network was constructed to present a 50 ohm load to the driving electronics. The spatial pressure distribution at the transducer focus and the pressure-voltage relationship were characterized in a hydrophone tank with a needle hydrophone (SN2344, Precision Acoustic, Dorchester, Dorset, UK). The transducer was connected to a custom-built driving system and stereotaxic apparatus described previously by our group^1^. Compared to the previous system, the increased transducer diameter required a water standoff to place the acoustic focus in the target region of the brain. This was achieved by constructing a 3D-printed cone which could be filled with water. A schematic of the system and photographs of the components are shown in Fig. 1a.

**Preparation of and description of the nanobubbles**

The nanobubbles were prepared by mixing a variety of lipids: DBPC (1,2-dibehenoyl-sn-glycero-3-phosphocholine, Avanti, Alabaster, AL, USA), DPPA (1,2 dipalmitoyl-sn-glycerol-3-phosphate, Corden Pharma, Plankstadt, Germany), DPPE (1,2-dipalmitoyl-sn-glycero-3-phosphoethanolamine, Corden Pharma, Plankstadt, Germany) and mPEG-DSPE (1,2-distearoyl-sn-glycero-3-phosphoethanolamine-N-[methoxy(polyethylene glycol)-2000], Laysan Bio, AL, USA) with a mass ratio of 6.15:1:2:1 in chloroform in a 1 ml vial^2^. Following solvent evaporation, hydration and gas exchange (to octafluoropropane), the bubbles were activated via mechanical agitation and centrifugation was performed to eliminate larger bubbles over 1 micron in size. The mean bubble hydrodynamic diameter was measured in MQ water with 1:1000 dilution with a particle size analyzer (Zetasizer Nano Range, Malvern Instruments Ltd, Worcestershire, UK) and was 313±13.2 nm. Particle concentration was 1.14 × 10^11^ ± 3.60 × 10^11^ /ml as measured using resonant mass measurement (Archimedes, Malvern Instruments Ltd, Worcestershire, UK) equipped with a nanosensor.

**In-vitro frequency response characterization**

For each of the formulations, the frequency spectrum of acoustic emissions from stimulated bubbles was evaluated as a function of transmitted focal pressure. The samples were prepared with a total gas volume of 1.1-1.2 µl/ml. First, a pressure sweep sonication was performed. The ultrasound focal pressure started from 0.21 MPa then increased to 1.13 MPa with the step size of approximately 0.01-0.03 MPa. Three acquisitions were acquired at each pressure level from which the mean and standard deviation were calculated.

In order to test the dependency on local concentration, the frequency response as a function of gas volume was characterized. Constant pressure sonication was performed in this test. According to the preliminary experiment results, different increasing trends were observed for three bubbles. To get an accurate reading of the corresponding frequency response with various gas volume, the peak negative focal pressure was fixed at its maximum value of 1.13 MPa for this experiment. Five samples with different gas volume for each of the three agents were prepared according to Supplementary Table S2 and measured with infusion administration. Pure saline was infused and measured at the same infusion rate as a baseline reference.

Persistence, defined as the time required to reduce the harmonic response from the stimulated bubbles to baseline levels, was another characteristic evaluated for all three agents. The frequency response of each agent decreases over time as each successive transmitted ultrasound pulse destroys a fraction of the bubble population. The persistence time depends on both the gas volume and size distribution of the bubble solution. With the focal pressure fixed at 1.13 MPa, successive ultrasound bursts were delivered to the tube while the flow was stationary, and the frequency response from the stimulated bubbles was recorded. Five samples with different gas volume were measured for each agent.

**Animal preparation**

Animals were anesthetized through inhalation using a mixture of 2-3% isoflurane and 1-2 L/min of 100% oxygen. A 24G I.V. catheter was placed in the lateral tail vein for drug administration. A pulse oximeter was used to monitor vital signs, and a temperature controlling and vital sign monitoring system (PhysioSuite, Kent Scientific Corp., Torrington, CT, USA) was used to monitor and maintain core body temperature. Hair over the cranial surface of the skull was removed using an animal trimmer and depilatory cream (VEET sensitive formula, Reckitt Benckiser, Parsippany, NJ, USA) to enable ultrasound propagation into the brain. After preparation, the animal was transferred to the stereotaxic apparatus and stabilized using ear bars and a bite bar. A custom-built nose cone was placed over the animal’s nose to deliver inhalant anesthetic during sonications.

A small skin incision was made over the skull to identify cranial landmarks for atlas registration. The registration procedure was performed using the method described previously^1^. Brain targets were selected in the region of the hippocampus on both hemispheres. Nine rats were used for primary in-vivo evaluation of the system. Twenty-five rats were used for in-vivo characterization and evaluation of three different agents (10 for Optison, 7 for Definity, 8 for nanobubble) and 6 rats were utilized for performing microscopy (4 for Optison, 1 for Definity, 1 for nanobubble). Evans blue (2%, 3.33ml/kg dosage) was injected via the tail vein catheter and allowed to circulate for a minimum of 3-5 minutes. Bubbles were administered through the tail vein catheter according to different sonication protocols. Animals were sacrificed using transcardiac perfusion with saline and 10% buffered formalin approximately 5-10 minutes after sonication. The brain was harvested immediately and placed in formalin for final fixation.

To evaluate the BBB opening effect with feedback controlled sonication, 2 targets were selected in each animal for ultrasound delivery. After brain atlas registration based on the current experimental animal, the coordinates of the landmark (Bregma) was set to be (0, 0, 0) along anterior-posterior (AP), medial-lateral (ML), and dorsal-ventral (DV) direction. The two targets were assigned to be (-5.2, 4.5, 3.0) in right hemisphere and (-5.2, -4.5, 3.0) in left hemisphere.

**Microscopy**

For immunofluorescence staining, slices were dried for 20 minutes at 37°C and fixed with 4% paraformaldehyde for 10 minutes. Slices were then placed in boiling citric acid buffer for antigen retrieval followed by washing in 1x PBS. Slides were blocked for 1 hour in 3% normal goat serum and incubated in primary antibody for RECA-1 (Ab9774, Abcam, Cambridge, MA,) for 1 hour at room temperature. Slides were then rinsed in PBS and incubated in DyLight 488 AffiniPure (1:300, #**212-485-168,** Jackson Immuno Research, West Grove, PA) secondary antibody for 2 hours, rinsed in PBS, and coverslipped with VectaSheild Mounting Medium with DAPI (Vector labs, Burlingame, CA). Images were taken at 40x magnification on the Zeiss Axio-Imager microscope and adjusted using Fiji^3^ by Image J.

**Statistical Methods**

ANOVA model was performed to evaluate the mean difference among three types of bubbles. For pressure sweep, a three-phase segmented linear regression model was used to model the changes in AUC level associated with the varying focal pressure. The correlation between AUC level and focal pressure can be interpreted as a low phase, a rising phase and a high phase connected by breakpoints. All breakpoints were estimated using least square estimation and t-tests were used to compare the difference in the first breakpoint across three agents. A larger breakpoint indicates a higher pressure is required for the bubble to enter a rising phase. In feedback control study, linear mixed models were used to test 1: the difference among three agents in standard deviation of AUC during controlling period, 2: the difference in mean pressure required for three agents during controlling period. In all statistical results, p-value less than 0.05 was considered as statistical significant difference. Tukey adjustment for multiple comparison was used when appropriate. All analysis was performed in SAS 9.4 (SAS Institute Inc., Cary, NC).

**Reference**

1. Bing, C. *et al.* Trans-cranial opening of the blood-brain barrier in targeted regions using a stereotaxic brain atlas and focused ultrasound energy. *J. Ther. ultrasound* **2,** 13 (2014).

2. Hernandez, C. *et al.* Ultrasound signal from sub-micron lipid-coated bubbles. in *2017 IEEE International Ultrasonics Symposium (IUS)* 1–4 (IEEE, 2017). doi:10.1109/ULTSYM.2017.8091670

3. Schindelin, J. *et al.* Fiji: an open-source platform for biological-image analysis. *Nat. Methods* **9,** 676–682 (2012).
